# Molecular method for the characterization of *Coxiella burnetii* from clinical and environmental samples: variability of genotypes in Spain

**DOI:** 10.1186/1471-2180-12-91

**Published:** 2012-06-01

**Authors:** Isabel Jado, Cristina Carranza-Rodríguez, Jesús Félix Barandika, Cristina García-Amil, Beatriz Serrano, Margarita Bolaños, Horacio Gil, Raquel Escudero, Ana L García-Pérez, A Sonia Olmeda, Ianire Astobiza, Bruno Lobo, Manuela Rodríguez-Vargas, José Luis Pérez-Arellano, Fernando López-Gatius, Francisco Pascual-Velasco, Gustavo Cilla, Noé F Rodríguez, Pedro Anda

**Affiliations:** 1 Laboratorio de Espiroquetas y Patógenos Especiales, Department of Bacteriology, Centro Nacional de Microbiología, Instituto de Salud Carlos III, Ctra. de Pozuelo km 2.6, Majadahonda, Madrid, 28220, Spain; 2 Microbiology Service and Infectious and Tropical Diseases Unit, Hospital Universitario Insular de Gran Canaria, Av Marítima del Sur, s/n, 35016, Las Palmas, Spain; 3 Department of Production and Animal Health, NEIKER - Instituto Vasco de Investigación y Desarrollo Agrario, Berreaga Kalea, 1, Derio, Bizkaia, 48160, Spain; 4 Department of Animal Health, Facultad de Veterinaria, Universidad Complutense de Madrid, Avda. Puerta de Hierro, s/n, Madrid, 28040, Spain; 5 Department of Animal Production, Universidad de Lleida, Pl. de Víctor Siurana, 1, Lleida, 25003, Spain; 6 Internal Medicine Service, Hospital Comarcal de Laredo, Avda. de los Derechos Humanos, s/n, Laredo, Cantabria, 39770, Spain; 7 Hospital Universitario Donostia and CIBERES, P° Doctor José Beguiristain s/n, Donostia-San Sebastián, Guipúzcoa, 20014, Spain; 8 Department of Animal Medicine and Surgery, Facultad de Veterinaria, Universidad de Las Palmas de Gran Canaria, Campus Universitario de Arucas s/n, Arucas, Las Palmas, 35413, Spain; 9 Present Address: Department of Molecular Genetics and Microbiology, Center for Infectious Diseases, Stony Brook University, Stony Brook, NY, 11794-5120, USA

## Abstract

**Background:**

*Coxiella burnetii* is a highly clonal microorganism which is difficult to culture, requiring BSL3 conditions for its propagation. This leads to a scarce availability of isolates worldwide. On the other hand, published methods of characterization have delineated up to 8 different genomic groups and 36 genotypes. However, all these methodologies, with the exception of one that exhibited limited discriminatory power (3 genotypes), rely on performing between 10 and 20 PCR amplifications or sequencing long fragments of DNA, which make their direct application to clinical samples impracticable and leads to a scarce accessibility of data on the circulation of *C. burnetii* genotypes.

**Results:**

To assess the variability of this organism in Spain, we have developed a novel method that consists of a multiplex (8 targets) PCR and hybridization with specific probes that reproduce the previous classification of this organism into 8 genomic groups, and up to 16 genotypes. It allows for a direct characterization from clinical and environmental samples in a single run, which will help in the study of the different genotypes circulating in wild and domestic cycles as well as from sporadic human cases and outbreaks. The method has been validated with reference isolates. A high variability of *C. burnetii* has been found in Spain among 90 samples tested, detecting 10 different genotypes, being those *adaA* negative associated with acute Q fever cases presenting as fever of intermediate duration with liver involvement and with chronic cases. Genotypes infecting humans are also found in sheep, goats, rats, wild boar and ticks, and the only genotype found in cattle has never been found among our clinical samples.

**Conclusions:**

This newly developed methodology has permitted to demonstrate that *C. burnetii* is highly variable in Spain. With the data presented here, cattle seem not to participate in the transmission of *C. burnetii* to humans in the samples studied, while sheep, goats, wild boar, rats and ticks share genotypes with the human population.

## Background

*Coxiella burnetii* is an obligate intracellular Gram negative bacterium which causes Q fever, an illness with multiple clinical manifestations in its acute presentation, including a flu-like respiratory process that could result in atypical pneumonia, or fever of intermediate duration (FID) with liver involvement. In a low percentage of cases a chronic form of the disease is diagnosed, characterized by an infection that persists for more than 6 months, more frequently endocarditis, which can be fatal without an appropriate treatment [1]. Its high infectivity, resistance in adverse environmental conditions and aerosol route of transmission make this agent a candidate for intentional release [2], being listed as a category B bioterrorism agent by the USA Centers for Disease Control and Prevention.

Initial studies tried to correlate specific genotypes (GT) with the chronic and acute forms of the disease. Thus, certain plasmid patterns were claimed to be associated with the disease outcome [3,4], which was controversial [5]; also, some *isocitrate dehydrogenase* types were associated with chronic disease and a role for this gene in the adaptation of the organism to the intracellular environment was proposed [6], although this association was also challenged by other authors [7].

More recently, different attempts have been made to classify isolates of *C. burnetii* in different genomic groups (GG). Based on restriction fragment length polymorphism (RFLP) of the entire genome, Hendrix et al. [8] resolved 36 isolates of different origin in 6 GG; Jager et al. [9] performed pulsed field gel electrophoresis (PFGE) in 80 isolates that were classified into 4 GG; a Multispacer Sequence Typing method [10], based on the sequencing of 10 intergenic spacers classified 173 isolates, mainly from chronic disease, into 3 monophyletic groups and 30 GT; later, a reduced MST method was published by Mediannikov et al. [11], targeting 3 spacers in a single PCR, detecting 3 MST GTs; Svraka et al. [12] amplified different variable number tandem repeats (VNTR) in 7 PCR protocols, describing 5 clusters and validating previous GG ascriptions; Arricau-Bouvery [13] applied infrequent restriction site-PCR and multilocus variable number of tandem repeats analysis (MLVA) for the typing in a scheme of 17 PCR protocols leading to a classification into 6 clusters and 36 GT; later, Roest et al. [14], used the same method but reducing the 17 described targets to 10, to study an outbreak in the Netherlands and describing 13 MLVA types; Beare et al. [15] added two more GG, totalling up to 8, in a microarray-based whole genome comparison; Denison et al. [16] performed 20 PCRs for the characterization of the region within and near the transposase *IS1111*, describing 5 GG among 21 reference strains and 9 animal samples; Huijsmans et al. [17] developed a method for a single-nucleotide-polymorphisms (SNP)-based typing, applying 10 real time PCR protocols that resolved 28 reference strains and 40 samples from an outbreak into 9 SNP genotypes, while a previous study on the same 28 reference strains [13] had disclosed 14 MLVA types; finally, Hornstra et al. [18] performed 14 SNP-based real time PCR assays that classified 63 isolates into 6 GG and 35 MST genotypes.

Recently, an outer membrane protein-coding gene named *acute disease antigen A* (*adaA*) was described as associated with acute Q fever-causing strains, whereas *adaA* negative strains were linked to chronic cases [19]. Therefore, the hypothesis of its association with a specific clinical presentation of the disease together with its immunodominant nature lead the authors to suggest that *adaA* may be a virulence factor for the pathogenesis of Q fever. Consequently, *adaA* may be a relevant genetic marker for differentiation among isolates.

In general, there has been a good correlation between typing methods although with different discriminatory capabilities. However, although 2 previous descriptions have been applied directly to clinical samples [16,17], both rely on the amplification of several targets performing between 10 and 20 PCR protocols, which make it not always feasible for their use in a clinical setting due to the frequent scarcity of testable sample-size, which hampers the acquisition of global data; the method of Mediannikov et al. [11], consisting of a multiplex PCR targeting 3 intergenic spacers, exhibited however a limited discriminatory power (3 MST types) in the samples studied. In this study, based on the previous descriptions of Beare et al. [15] and Zhang et al. [19], a fast, reproducible and sensitive multiplex PCR that amplifies 8 targets in the same run for a rapid GT determination, has been developed to be applied to both isolates and PCR-positive samples. With this method, *C. burnetii* could be classified into 8 GG and up to 16 GT. Based on this methodology, a comprehensive study on the variability of *C. burnetii* in Spain have been made with samples from patients with acute and chronic Q fever, domestic and wild mammals and ticks, demonstrating a high variability of this organism and an association between genotypes and human disease.

## Methods

### Samples

Fifteen *C. burnetii* reference isolates were used to validate the typing method (Additional file 1: Table S1). Also, human and animal samples (livestock, wild animals and ticks) sent to the National Reference Laboratory at the Instituto de Salud Carlos III and to the clinical and veterinarian collaborating laboratories for diagnosis of Q fever were included in the study, including defibrinated blood, plasma, biopsy material, ruminant placentas, mostly from abortions with the exception of 3 cattle placentas from normal parturitions (Additional file 1: Table S1), and other tissues from domestic and wild animals, and questing ticks, that were collected from different areas in Central Spain: 4 areas in Madrid (Cercedilla, Aranjuez, Perales and Valdeolmos) and 1 in Toledo (Oropesa). In all the areas the presence of livestock was documented (cattle in all areas and sheep and swine only in Oropesa). There were remarkable high densities of rabbits (*Oryctolagus cuniculus*) in all the areas except Cercedilla. The study protocol was approved by the Bioethics and Animal Welfare Committee of the Instituto de Salud Carlos III, Spain (ref. CBBA/4 2006), where the study was conducted, respecting individual privacy according to relevant data protection legislation and animal welfare. Also, human clinical samples used in the study were made available to us in an anonymized manner.

### Culture

Standard shell-vial methodology was used as previously described [20] to grow *C. burnetii* in Vero E6 cells (European Collection of Cell Cultures; provided by Sigma-Aldrich Química S.A., Tres Cantos, Madrid, Spain). All the propagative methods and those related to the manipulation of domestic ruminant placentas were performed under Biosafety level 3 (BSL3) conditions.

### Molecular detection of *C. burnetii*

DNA was extracted from samples and isolates with the Qiagen Tissue kit (IZASA S.A. Barcelona, Spain). For arthropods, specimens were first crushed in 1.5 ml eppendorf tubes with the help of a pestle (Sigma-Aldrich Química S.A., Barcelona, Spain), as described [21], and extracted as before. DNA was quantified in a NanoDrop ND-1000 spectrophotometer (NanoDrop Technologies Inc. Wilmington, Delaware USA), and about 200 ng were used for each PCR. Previous to the genotyping, a screening assay (*IS1111-*based PCR coupled with hybridization with a specific probe by reverse line blotting -RLB) was used for the detection of *C. burnetii*[22-24].

### *C. burnetii* genotyping

An analysis based on a previous report [15] was performed to identify which genes/ORFs defined the ascription of each isolate to a specific GG, and seven of them were selected (CBU0007, CBU0071, CBU0168, CBU0598, CBU0881, CBU1805 and CBU2026), whose combination of presence/absence seems to determine the GG (Table 1). Also, the detection of *adaA* (CBU0952) [19] was included in the method. Primers and probes were designed for their molecular detection by PCR and subsequent RLB hybridization (Additional file 2: Table S2) by using Oligo6 software (Molecular Biology Insights, Inc. Cascade, CO, USA). Incompatibility among primers was avoided by *in silico* analysis of the formation of secondary structures, and oligonucleotides forming dimers with energy values lower than −6 kcal/mol and hairpins with Tm higher than 40C were discarded. The specificity of the oligonucleotides was first assessed by blastn (http://www.ncbi.nlm.nih.gov/blast/Blast.cgi?PAGE=Nucleotides). The reaction mix included 80 μg/tube of bovine serum albumin (Roche España, Madrid, Spain), 3.75 mM MgCl_2_ (Applied Biosystems), 200 μM dNTPs (Applied Biosystems) and 4U of AmpliTaq Gold® DNA Polymerase (Amersham Pharmacia Biotech, Cerdanyola del Vallès, Barcelona, Spain). Primer concentrations ranged from 0.6 to 1 μM (Additional file 2: Table S2). The amplification cycles included an initial cycle of 94C for 9 min, followed by 40 cycles of 94C 30 s, 60C 1 min, and 72C 1 min, with a final extension at 72C for 10 min. The amplifications were performed in an MJ Research PTC-200 (Bio-Rad Laboratories, S.A., Alcobendas, Madrid, Spain) in volumes of 50 μl. Hybridization by RLB was performed as described [25] using 48C for the hybridization and 40C for the conjugate and the washing steps. Concentration of probes ranged from 0.8 to 6.4 pmols/μl (Additional file 2: Table S2). Two overlapping films (SuperRX, Fujifilm España S.A., Barcelona, Spain), were used in each assay to obtain a less and more exposed image for each membrane.

**Table 1:** **Scheme of the presence/absence of the*Coxiella burnetii*ORFs selected for the determination of genomic groups**

**Target**	**GGI**	**GGII**	**GGIII**	**GGIV**	**GGV**	**GGVI**	**GGVII**	**GGVIII**
**CBU0007**	+	+	+	−	+	+	+	+
**CBU 0071**	+	+	+	+	−	+	+	−
**CBU 0168**	+	+	+	−	+	+	−	+
**CBU 0598**	+	+	−	+	+	+	+	+
**CBU 0881**	+	+	+	+	+	−	−	−
**CBU 1805**	+	+	+	+	−	+	+	+
**CBU 2026**	+	−	+	+	+	+	+	+

The sensitivity of the technique was checked with serial 10-fold dilutions of a purified DNA stock of the isolate Nine Mile phase II (NMII) and the specificity was studied by subjecting to the method 10^4^ genome equivalents of a selection of other bacterial species causing zoonoses or related illness (*Orientia tsutsugamushi, Rickettsia conorii, R. typhi, Legionella pneumophila, Francisella tularensis* subsp*. holarctica, Bartonella henselae, Chlamydophila pneumoniae,* and *Mycoplasma pneumoniae*).

To assess the reproducibility of the methodology, DNA extracted from 2 different passages (n and n+10) of 5 reference isolates (NMI, CS-27, Priscilla, SQ217, F2) and a local isolate from cattle (273) (Additional file 1: Table S1) were analyzed.

The results of the GT study were further analyzed by using InfoQuest™FP 4.50 (BioRad, Hercules, CA, USA). Clustering analyses used the binary coefficient (Jaccard) and UPGMA (Unweigthed Pair Group Method Using Arithmetic Averages) to infer the phylogenetic relationships.

## Results

### Samples included in the study

A total of 90 autochthonous samples were included: 24 from human patients (11 from acute and 13 from chronic cases) and 66 from animals and ticks (15 from sheep, 7 from goats, 7 from cattle, 3 from rats, 1 from a wild boar and 33 from ticks); also, 15 reference strains were used to validate the method (Additional file 1: Table S1).

### Sensitivity, specificity and reproducibility of the method

**Figure 1:**
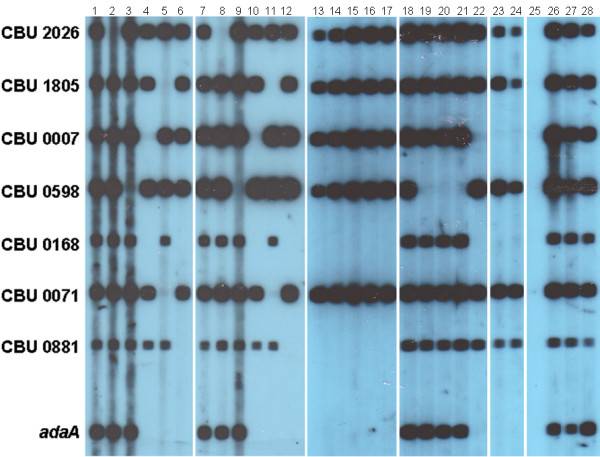
**Sensitivity and reproducibility of the method of characterization of*Coxiella burnetii.*** Reproducibility. Lanes 1–6: Isolates NMI, CS-27, local cattle isolate 273, Priscilla, SQ217 and F2, passage “n”; lanes 7–12: same isolates, passage “n+10”. Results using clinical, veterinary and arthropod samples. Lane 13: specimen of *R. sanguineus* (M28CE4GA7C); lanes 14–16: specimens of *H. lusitanicum* (M28P1GA8A, M28PE14GV5C and M28PE14GV5F); lane 17: specimen of *D. marginatus* (M28P2GA45C); lane 18: human serum (2172); lane 19: sheep placenta (70924); lane 20: goat lung (67025); lane 21: cattle endocervical exudate (70814); lane 22: human clot (BZO18); lane 23: human plasma (0904); lane 24: rat liver (78); lane 25: negative control. Sensitivity. Lanes 26–28: 10^3^, 10^2^ and 10 genome equivalents of isolate NMII. Left panel: position of the probes for each ORF.

The detection limit of the method using purified NMII DNA was 10 genome equivalents. No reactivity was detected when testing 10^4^ genome equivalents from 8 other bacterial species causing zoonoses or related illness (data not shown). To check for reproducibility, passages “n” (Figure 1, lanes 1–6) and “n+10” (Figure 1, lanes 7–12) from 5 reference isolates and a local isolate from cattle were checked without any loss of sensitivity during *in vitro* passages in any of the targets assayed. Also, it is to note that NMI (phase I) and NMII (phase II) isolates presented the same results in this characterization; consequently, only one of them (NMI) was used throughout the study.

### Genotyping of reference isolates and samples

**Figure 2:**
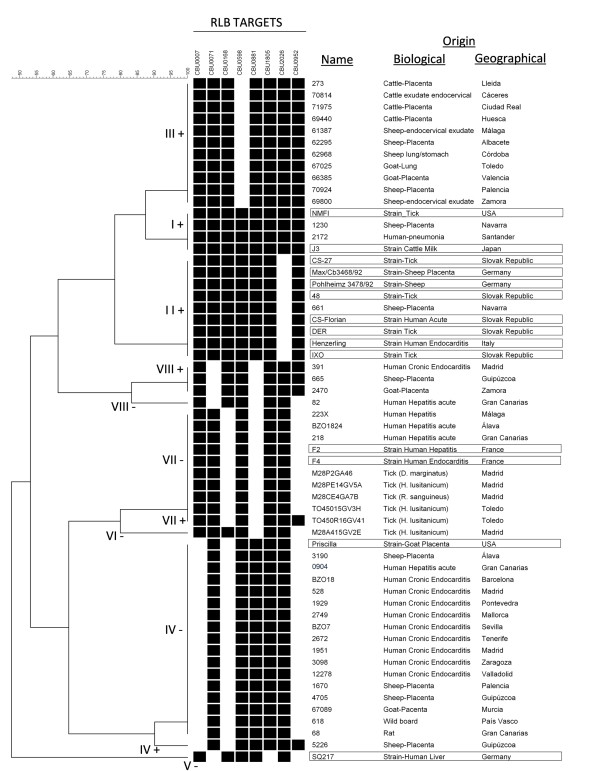
**Dendogram construct from hibridization data of 58 local samples and reference strains**. Biological and geographical origin of the samples is shown. Black boxes indicate presence of the selected ORFs; reference isolates used to validate the method are framed.

From the 15 reference isolates that were tested with the method described here for GG adscription (Table 1), and from which data from previous studies were available, all of them fell in the same GG as previously described, the topology of the tree being consistent with previous data. None of the reference isolates tested was found to belong to GG III, VI and VIII (Additional file 1: Table S1, Figure 2).

**Figure 3:**
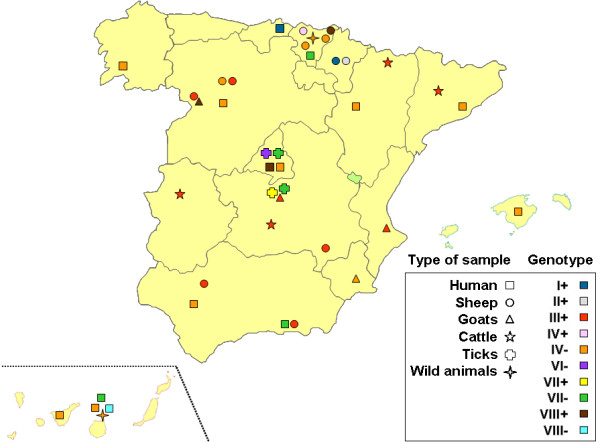
Map of Spain showing sampling sites, type of samples and results.

Local human samples were found to belong to GG I, IV, VII and VIII, as follows: 13 samples from chronic cases (7 endocarditis, 3 vascular infections, 1 infected aneurism, 1 osteomyelitis and 1 chronic hepatitis) from 8 different regions were all infected with GG IV, except for one vascular infection (GG VIII); acute cases (10 samples of FID with liver involvement and 1 sample of pneumonia; 4 regions) showed GG I, IV, VII and VIII (Additional file 1: Table S1, Table 2, Figures 2 and 3).

**Table 2:** Summary of the results of the characterization

**GT**	**Human samples**			**Livestock**					
	**Acute hepatitis**	**Acute pneumonia**	**Chronic**	**Sheep**	**Goat**	**Cattle**	**Wild animals**	**Ticks**	**Total**
I+*		1		1					2
II+				1					1
III+				5	3	7			15
IV+				1					1
IV-	6		12	5	1		4		28
VI-								1	1
VII+								1	1
VII-	3							31	34
VIII+			1	2	3				6
VIII-	1								1
Total	10	1	13	15	7	7	4	33	90

*: plus and minus signs refer to *adaA* presence/absence, respectively.

Among livestock samples, those from sheep (15 samples from 8 provinces) were found belonging to GG I, II, III, IV and VIII; goats (7 samples from 4 provinces) were infected with GG III, IV and VIII; cattle (7 samples from 4 provinces) were all infected by GG III; rats (3 samples from 1 province) and a wild boar showed GG IV; finally, 33 ticks of 3 species, from 4 areas of 2 adjacent regions, carried always GG VII, except for one that carried GG VI. In summary, samples from GG I, II, III, IV, VI, VII and VIII were identified (Additional file 1: Table S1; Table 2, Figures 2 and 3).

### *adaA* detection

Samples from GG I, II and III were always *adaA* positive; all GG IV were *adaA* negative, except for a sheep placenta that was *adaA* positive; GG VII samples were *adaA* negative, except for a tick specimen; GG VIII samples were positive, except for a human sample of acute hepatitis; finally, the only sample available from GG VI (one *H. lusitanicum* tick) was *adaA* negative (Additional file 1: Table S1, Table 2, Figure 2). All the samples from cases of acute FID with liver involvement (10 samples from 3 distant regions; Figure 3) were *adaA* negative and the only sample available from a patient with pneumonia was *adaA* positive.

In summary, from the theoretically possible 16 GT (8 GG positive or negative for *adaA*), 10 were identified in the samples studied (Table 2).

## Discussion

A multiplex PCR coupled with hybridization by RLB for the characterization of *C. burnetii* was designed, allowing for its classification into the previously known 8 GG [15] and into up to 16 genotypes, depending on *adaA* presence/absence. For validation, 15 reference strains characterized in previous studies were used (Additional file 1: Table S1). All of them fell in the same GGs as previously described, when data was available, or grouped in the same clade as described [8-10,12,13]. Consequently, an excellent correlation with some previously published schemes and, specifically, with the microarray-based whole genome typing of Beare et al. [15] was observed: the 4 isolates studied by Beare et al. that were also analyzed in this study (NMI, GG I; Henzerling, GG II; Priscilla, GG IV; and Scurry Q217, GG V) were classified with this method into the same GG as described. Also, the analysis of the results by InfoQuest disclosed a tree whose topology was similar to that of Beare et al. [15], with the only exception of GG VIII, which in this study grouped together with GGs I, II and III, instead of with GGs IV, V, VI and VII as in the Beare study.

With this methodology, a preliminary characterization of *C. burnetii* variants circulating in Spain has been performed showing a high variability of this organism in clinical and environmental settings, identifying 7 GG, with the exception of GG V, and 10 different GTs.

In Spain, while a respiratory disease is observed in about 80% of cases reported from the Northern region of the Basque Country [26,27], the Southern regions of Andalusia and the Canary Islands report a clear predominance (about 90% of cases) of FID with liver involvement [28-34]. This last has also been described in Australia, France, Greece, or Taiwan [35-38], among others. Even taking into account the limited size of this study and the constraint of an extrapolation, a strain-associated factor that might explain the different clinical presentations of acute Q fever is hypothesized for our country. The pattern observed in cases of acute Q fever indicates an association between absence of *adaA* and FID with liver involvement, produced in this study by *adaA* negative strains in both regions (the Southern regions of Andalusia and the Canary Islands), although is not statistically significant in this study (*p* = 0.09) due to low number of samples. Also, another sample of a case of hepatitis from the north (Basque Country) yielded an *adaA* negative result as well. The same applies for the 2 reference isolates from hepatitis cases analyzed in this study: F2, a French isolate and SQ217, recovered in the USA from a case of chronic hepatitis, are both *adaA* negative as well. In contrast, pneumonia predominates over liver involvement in Northern Spain, being the only case of this clinical form available for the study produced by an *adaA* positive strain. No other marker used in this study correlated with the clinical presentation of acute Q fever. Availability of samples from cases of acute Q fever for genotyping is much less frequent than from cases of chronic Q fever, even though acute Q fever is much more prevalent. In this study, 11 samples from acute cases were analyzed, although only one was from a case with respiratory symptoms, reflecting the limited availability of such samples, which may be due to a poor clinical awareness.

From the 10 GTs found in the country, only 5 have been detected in humans and, among them, GT IV- is the most frequently found in acute and chronic cases (75% of cases). This GT has also been found in many mammal species (sheep, goat, wild boar and rats). Whether this could be interpreted as a higher tendency of this GT to cause illness in humans can not be inferred by this study, mainly considering that most of the acute cases (8/11) came from the same area (Gran Canaria Island). In any case, GT IV- is highly prevalent also in our chronic cases that came from 8 distant areas of the country, showing a more intensive circulation of this GT in humans.

The association previously proposed between *adaA-*negative strains and chronic disease [19] has been reproduced here for all chronic cases studied (13 cases), except for a sample from a vascular infection that was *adaA* positive. Beare et al. [15] hypothesized that the association between GG IV and chronic cases (as in 12 out of 13 chronic cases studied here) could be related to the slow growth of isolates from this genotype and, therefore, the induction of a decrease in the immune response. On the other hand, Zhang et al. hypothesized that *adaA* positive strains were related to acute cases [19], as it is the case of the only sample from a patient with acute pneumonia available. However, in our study, acute cases of FID with liver involvement were all produced by *adaA* negative strains.

GTs found in humans were also found in sheep, goats, rats, wild boar and ticks. This distribution of GTs suggests that sheep and goats are responsible for the transmission of *C. burnetii* to humans in Spain, as in other areas [39], and exhibit a high variability of GT. However, although in general domestic ruminants are important reservoirs for *C. burnetii* and play a relevant role in its transmission to humans, 4 of 24 human samples were found carrying GTs not found in ruminants in this work. A recent Spanish study [40] has also detected *C. burnetii* in roe deer, wild boar, carrion birds and hares. Although there is no data available on the genotyping of these specimens, more studies are needed to characterize the enzootic cycle of *C. burnetii* and its GT distribution in wildlife, as well as to ascertain whether other sources could be responsible for the transmission of *C. burnetii* to humans. GG VII was only found in ticks (*H. lusitanicum, Dermacentor marginatus* and *Rhipicephalus sanguineus*) and in 3 cases out of 10 of FID with liver involvement. It is to note that, while reference isolates from ticks belonged mostly to GG II, this GG has not been found in ticks in our study. Although the analyzed tick specimens came from 5 different areas, they were all from Central Spain, which could be biasing this data. Transmission of Q fever by tick bite still remains controversial [41,42], and cases of simultaneous or consecutive infections with *C. burnetii* and other tick-borne agents have been described [43]. Whether *C. burnetii* can be transmitted by tick bite or not, the detection in ticks of GT VII-, found only in human patients revives this debate. More studies are needed to definitely clarify this question. On the other hand, given that GG VII isolates have not been found in cattle, sheep and goats in this study, we could think of other unknown reservoirs that could be involved as a source of infection of this GG for both ticks and humans. Traditional mammal species on which the tick species analyzed in this study feed on include rabbits (frequent all over Spain) for the immature stages of *H. lusitanicum,* which seems to be very important for the maintenance of populations of this tick species, small mammals for those of *D. marginatus,* and canids for all stages of *R. sanguineus*. Adult *H. lusitanicum* and *D. marginatus* normally feed on large ungulates. Animals present in the tick study areas included, apart from cattle, high densities of rabbits and other wildlife. It is to note that 40 liver samples from rabbits hunted in Gran Canaria analyzed by PCR were all negative (data not shown), although more studies are needed. Whether some of the above mentioned animals may act as reservoirs for GG VII *C. burnetii* remains to be studied.

Interestingly, in 7 cattle samples from 4 distant regions, only GG III was detected. In the study of Arricau-Bouvery [13] most of the cattle isolates (12/14) analyzed by MLVA also grouped together in a clade that is close but different to the one that include GG I isolates, as in this study. In Beare’s study GG III is also philogenetically close to GG I and both clades appear together in the tree. This GG having never been found in humans in Spain so far lead us to hypothesize that cattle could represent a low risk for Q fever transmission to humans in our country.

One of the added values of the method described here is that it could be applied to any PCR-positive sample carrying at least 10 genome equivalents of the target organism, thus avoiding the need for culturing the organism to obtain data on the global circulation of *C. burnetii*. The frequent lack of human isolates from outbreaks, which are needed to apply the yet described methods, hamper a correct outbreak study that are necessary to identify the source of infection. This methodology allows the characterization directly from clinical samples avoiding the culture step of this fastidious bacterium, and proves to be valuable identifying so far 10 different GTs circulating in Spain. This method can be performed in any laboratory with basic equipment. It can easily determine relationships among *C. burnetii* from different origins by using PCR-positive samples, thus helping in the identification of the source of an outbreak in a rapid analysis.

## Conclusions

The method described here is rapid, reproducible and sensitive. It can be applied directly to clinical and environmental samples, and is able to identify up to 16 GT. This will facilitate the acquisition of global data on the circulation of GT of this organism.

We have found a high variability of *C. burnetii* in Spain, with 10 GTs found in different settings, 5 of them in human samples. Interestingly, all the samples from acute cases of FID with liver involvement were produced by *adaA* negative microorganisms, while the only case of pneumonia available for the study was caused by a *adaA* positive strain. Moreover, the majority (12 cases) of the 13 chronic cases studied were produced by organisms of GG IV-, except for a case of vascular infection (GG VIII +).

Regarding livestock, human cases share GTs with sheep and goats, but the only GT found in cattle has never been found in humans.

The most frequent GT found in ticks (GT VII-; 31 out of 33 specimens studied) was also found in human samples of cases of FID with liver involvement. Although the hypothesis of transmission of Q fever by tick bite still remains controversial, to further study this point is of interest.

## Authors’ contributions

IJ, HG, RE and PA participated in the design of the study. CCR, MB, JLPA and NFR studied clinical and environmental samples from Canary Islands suspected of *C. burnetii* infection and provided the positives to the Centro Nacional de Microbiología-Instituto de Salud Carlos III (CNM-ISCIII) for molecular analysis. JFB, IA and ALGP studied livestock and tick samples from the Basque Country and provided the positives to the CNM-ISCIII for characterization. AT and ASO studied environmental samples from Madrid and provided the positives to the CNM-ISCIII for characterization. BS and FLG studied livestock samples from Catalonia and provided the positives to the CNM-ISCIII for molecular analysis. FPV and GC studied samples from Q fever patients and provided the positives to the CNM-ISCIII for molecular analysis. IJ, CGA and MRV participated in the culture and manipulation of the isolates in the BSL3 laboratory. IJ and PA designed the method of characterization. IJ, RE, CGA, BL and MRV evaluated and carried out the genotyping method. HG and PA performed the phylogenetic analysis. IJ, HG, RE and PA participated in the interpretation of data and drafted the manuscript. All authors have critically read and approved the final version of the manuscript and also concur with further revisions of it.

## Supplementary Material

Additional file 1 **Table S1.** Samples and reference isolates used in the study. Click here for file

Additional file 2 **Table S2.** Oligonucleotides used in the study. Click here for file
