# Supplementary material for: Molecular method for the characterization of Coxiella burnetii from clinical and environmental samples: variability of genotypes in Spain
Source: BMC Microbiol. 2012 Jun 1;12:91. doi: 10.1186/1471-2180-12-91 (PMC3413600; doi:10.1186/1471-2180-12-91)
Supplement: Additional file 1 — Table S1. Samples and reference isolates used in the study. [file 1471-2180-12-91-S1.doc]

### Additional file 1: Table S1 – Samples and reference isolates used in the study

| **Reference isolates** | **Geographic origin** | **Source (clinical signs)** | **Year** | **GG** | ***adaA*** |
| --- | --- | --- | --- | --- | --- |
| CS-27 | Slovak Republic | Tick | 1968 | II | + |
| 48 | “ | *Haemaphysalis punctata* tick | 1970 | II | + |
| CS-Florian | “ | Human blood | 1956 | II | + |
| DER | “ | *Dermacentor marginatus* | 1967 | II | + |
| F2 | France | Human blood (hepatitis) | ND | VII | - |
| F4 | “ | Human blood (endocarditis) | ND | VII | - |
| Henzerling | Italy | Human blood (acute) | 1945 | II | + |
| IXO | Slovak Republic | *Ixodes ricinus* tick | 1957 | II | + |
| J3 | Japan | Cow’s milk | ND | I | + |
| Max/CB3468/92 | Germany | Sheep placenta | ND | II | + |
| NMFI and NMII | USA | *D. andersoni* tick | 1937 | I | + |
| PohlheimZ3478/92 | Germany | Sheep | ND | II | + |
| Priscilla | USA | Goat placenta | ND | IV | - |
| SQ217 | “ | Human liver (hepatitis) | 1981 | V | - |
| Z346(4)92 | Germany | Goat placenta | 1992 | II | + |
| **Samples studied** | **Province of Spain** |  |  |  |  |
| 2172 | Cantabria | Human serum (acute pneumonia) | 2005 | I | + |
| 223X | Málaga | Human liver biopsy (acute hepatitis) | 2004 | VII | - |
| BZO1824 | Álava | Human plasma (acute FID) | 2007 | VII | - |
| 0904 | Las Palmas | Human plasma (acute FID) | 2004 | IV | - |
| 82 | Las Palmas | “ | 2006 | VIII | - |
| 218 | “ | “ | 2007 | VII | - |
| 369 | “ | “ | 2009 | IV | - |
| 344 | “ | “ | 2009 | IV | - |
| 282 | “ | “ | 2008 | IV | - |
| 93 | “ | “ | 2006 | IV | - |
| 284 | “ | “ | 2008 | IV | - |
| BZO18 | Barcelona | Human clot (chronic, vascular infection) | 2005 | IV | - |
| 552 | “ | “ | 2006 | IV | - |
| 1951 | Madrid | Human vein biopsy (chronic, vascular infection) | 2005 | IV | - |
| 528 | “ | Human blood (chronic, endocarditis) | 2006 | IV | - |
| 3665 | “ | “ | 2005 | IV | - |
| 219 | “ | Human endocardium tissue (chronic, endocarditis) | 2001 | IV | - |
| 1929 | Pontevedra | Human endocardium tissue (chronic, endocarditis) | 2006 | IV | - |
| 2749 | Mallorca | Human valve biopsy (chronic, endocarditis) | 2006 | IV | - |
| 2672 | Tenerife | Human valve biopsy (chronic, endocarditis) | 2007 | IV | - |
| BZO7 | Sevilla | Human (chronic osteomielitis) | 2007 | IV | - |
| 3098 | Zaragoza | Human liver (chronic, hepatitis) | 2006 | IV | - |
| 391 | Madrid | Human aorta biopsy (chronic, vascular infection) | 2008 | VIII | + |
| 12278 | Valladolid | Human blood (chronic, endocarditis) | 2011 | IV | - |
| 665 | Guipúzcoa | Sheep placenta from abortion | 2008 | VIII | + |
| 68 | “ | “ | 2008 | VIII | + |
| 5226 | “ | “ | 2007 | IV | + |
| 4705 | “ | “ | 2000 | IV | - |
| 1670 | Palencia | “ | 2002 | IV | - |
| 70924 | “ | “ | 2010 | III | + |
| 3190 | Álava | “ | 2001 | IV | - |
| 4725 | “ | “ | 2002 | IV | - |
| 2984 | “ | “ | 2001 | IV | - |
| 661 | Navarra | “ | 2001 | II | + |
| 1230 | “ | “ | 2002 | I | + |
| 62295 | Albacete | “ | 2008 | III | + |
| 61387 | Málaga | Sheep endocervical exudate from abortion | 2008 | III | + |
| 69800 | Zamora | “ | 2010 | III | + |
| 62968 | Córdoba | Sheep Lung and stomach from abortion | 2009 | III | + |
| 2470 | Zamora | Goat placenta from abortion | 2005 | VIII | + |
| 24691 | “ | “ | 2005 | VIII | + |
| 24692 | “ | “ | 2005 | VIII | + |
| 66385 | Valencia | “ | 2009 | III | + |
| 71557 | Toledo | “ | 2011 | III | + |
| 67089 | Murcia | “ | 2010 | IV | - |
| 67025 | Toledo | Goat Lung from abortion | 2010 | III | + |
| 71975 | Ciudad Real | Cattle placenta from abortion | 2011 | III | + |
| 71859 | “ | “ | 2011 | III | + |
| 69440 | Huesca | “ | 2010 | III | + |
| 273 | Lleida | Cattle placenta from normal parturition | 2010 | III | + |
| 275 | “ | “ | 2010 | III | + |
| 286 | “ | “ | 2010 | III | + |
| 70814 | Cáceres | Cattle endocervical exudate from abortion | 2010 | III | + |
| 618 | Basque Country | Wild boar | 2001 | IV | - |
| 68 | Las Palmas | Wild rat spleen (*Rattus norvegicus*) | 2009 | IV | - |
| 78 | “ | “ | 2009 | IV | - |
| 81 | “ | “ | 2009 | IV | - |
| M28CE4GA7B | Madrid (Cercedilla) | *Rhipicephalus sanguineus* tick | 2004 | VII | - |
| M28CE4GA7C | “ | *R. sanguineus tick* | 2004 | VII | - |
| M28P2GA45B | Madrid (Perales) | *Dermacentor marginatus* tick | 2004 | VII | - |
| M28P2GA45C | “ | “ | 2004 | VII | - |
| M28P2GA45D | “ | “ | 2004 | VII | - |
| M28P2GA45F | “ | “ | 2004 | VII | - |
| M28P2GA45G | “ | “ | 2004 | VII | - |
| M28P2GA46 | “ | “ | 2004 | VII | - |
| M28AR15GV3 | Madrid (Aranjuez) | “ | 2004 | VII | - |
| M28A415GV2E | “ | *Hyalomma lusitanicum* tick | 2004 | VI | - |
| M28P1GA8A | Madrid (Perales) | “ | 2004 | VII | - |
| M28PE14GV3J | “ | “ | 2004 | VII | - |
| M28PE14GV5A | “ | “ | 2004 | VII | - |
| M28PE14GV5C | “ | “ | 2004 | VII | - |
| M28PE14GV5E | “ | “ | 2004 | VII | - |
| M28PE14GV5F | “ | “ | 2004 | VII | - |
| M28PE14GV5G | “ | “ | 2004 | VII | - |
| M28PE14GV5H | “ | “ | 2004 | VII | - |
| M28PE15GV4H | “ | “ | 2004 | VII | - |
| M28AL16GV1S | Madrid (Aranjuez) | “ | 2004 | VII | - |
| M28AL16GV2C | “ | “ | 2004 | VII | - |
| M28AL16GV2D | “ | “ | 2004 | VII | - |
| M28AL16GV2E | “ | “ | 2004 | VII | - |
| M28AL16GV2H | “ | “ | 2004 | VII | - |
| M28AL16GV2J | “ | “ | 2004 | VII | - |
| M28AL16GV2O | “ | “ | 2004 | VII | - |
| M28AL16GV2P | “ | “ | 2004 | VII | - |
| M28AL16GV2Z | “ | “ | 2004 | VII | - |
| M28AL16GV3B | “ | “ | 2004 | VII | - |
| TO45OR15GV3H | Toledo (Oropesa) | “ | 2004 | VII | - |
| TO45OR15GV3I | “ | “ | 2004 | VII | - |
| TO45OR15GV3M | “ | “ | 2004 | VII | - |
| TO45OR16GV4I | “ | “ | 2004 | VII | + |
