# Supplementary material for: Molecular method for the characterization of Coxiella burnetii from clinical and environmental samples: variability of genotypes in Spain
Source: BMC Microbiol. 2012 Jun 1;12:91. doi: 10.1186/1471-2180-12-91 (PMC3413600; doi:10.1186/1471-2180-12-91)
Supplement: Additional file 2 — Table S2. Oligonucleotides used in the study. [file 1471-2180-12-91-S2.doc]

**Additional file 2: Table S2 - Oligonucleotides used in the study**

| Target | Position in the genome  (RSA 493) | Size (pb) | Primers (5’-3’)^a^ | Final concentration  (µM) | Probes (5’-3’)^b^  (concentration pmols/µL) |
| --- | --- | --- | --- | --- | --- |
| CBU0007 | 8196-8398 | 247 | ATCAATACAAGAGGGTAATGCTATG | 1 | CTTGTATTTACGTGAGTGTGCG (0.8) |
|  |  |  | TAGCAAACTTGGCTCTATTTTTCCC | 1 |  |
| CBU0071 | 64571-65545 | 186 | CCCCAGCAGCCAATATCGCAGCG | 0.6 | GGATAAGAGGTCCCAGCTCG (6.4) |
|  |  |  | CGGCGTGGAACTTATCCTCTATC | 0.6 |  |
| CBU0168 | 153755-154033 | 208 | GGTTGCTCTAACAGATACTATACAG | 1 | CGCTAATTCAATGGATCGC (1.6) |
|  |  |  | GTCTAATAAATCTGCTAATTCACG | 1 |  |
| CBU0598 | 545874-546419 | 220 | CGGGTATGGATGAATATGTGTTG | 0.6 | CGCTTCTCCTGGCTATTTGC (0.8) |
|  |  |  | TCAGGTTCATCACCAACCATCG | 0.6 |  |
| CBU0881 | 833049-833714 | 159 | AGAGAAATGGCTAACTTGGGAAG | 1 | ATATTCTCTTGAGAGCGTATGA (3.2) |
|  |  |  | AGCTTCTCTTGAGTTGAAAATCC | 1 |  |
| CBU1805 | 1736367-1736750 | 268 | ACCACCAAAATCCCCACAGAAAC | 0.6 | CGCTACAAACAAATTGCTGAAG (0.8) |
|  |  |  | CCGTGGATGGTGACTCAAAATATC | 0.6 |  |
| CBU2026 | 1934417-19344722 | 306 | ATGAGGAGTGCCTTCAATAGTC | 0.6 | CGTTATTACACGGAAATTACATG (6.4) |
|  |  |  | CTACACGGTTAGTGGGAAGCGG | 0.6 |  |
| CBU0952 (*adaA*) | 299708-300391 | 172 | GTTGAAAAATGGGTAACGACGG | 0.6 | GCCGATGTGATGGTTTCCG (3.2) |
|  |  |  | CCGACTGATCCTGTGACATTG | 0.6 |  |

a: 5’ biotin modification in all the primers

b: 5’ C6 aminolink modification in all the probes
